# Factors related to intentional non-initiation of bisphosphonate treatment in patients with a high fracture risk in primary care: a qualitative study

**DOI:** 10.1186/s12875-018-0828-0

**Published:** 2018-08-23

**Authors:** Karin M. A. Swart, Myrthe van Vilsteren, Wesley van Hout, Esther Draak, Babette C. van der Zwaard, Henriette E. van der Horst, Jacqueline G. Hugtenburg, Petra J. M. Elders

**Affiliations:** 1 Department of General Practice and Elderly Care Medicine, Amsterdam Public Health research institute, Amsterdam UMC, Vrije Universiteit Amsterdam, De Boelelaan 1117, 1081 HV Amsterdam, Netherlands; 2 Stichting Artsen Laboratorium en Trombosedienst, Molenwerf 11, 1541 WR Koog aan de Zaan, Netherlands; 3 Department of Clinical Pharmacology and Pharmacy, Amsterdam Public Health research institute, Amsterdam UMC, Vrije Universiteit Amsterdam, De Boelelaan 1117, 1081 HV Amsterdam, Netherlands

**Keywords:** Bisphosphonates, Osteoporosis, Non-initiation, General practitioner

## Abstract

**Background:**

Adherence to osteoporosis treatment is crucial for good treatment effects. However, adherence has been shown to be poor and a substantial part of the patients don’t even initiate treatment. This study aimed to gain insight into the considerations of both osteoporosis patients and general practitioners (GP) concerning intentional non-initiation of bisphosphonate treatment.

**Methods:**

Osteoporosis patients and GPs were recruited from the SALT Osteoporosis Study and a transmural fracture liaison service, both carried out in the Netherlands. Using questionnaires, we identified non-starters and starters of bisphosphonate treatment. Semi-structured interviews were conducted to gain a detailed overview of all considerations until saturation of the data was reached. Starters were asked to reflect on the considerations that were brought forward by the non-starters. Interviews were open coded and the codes were classified into main themes and subthemes using an inductive approach.

**Results:**

16 non-starters, 10 starters, and 13 GPs were interviewed. We identified three main themes: insufficient medical advice, attitudes towards medication use including concerns about side effects, and disease awareness. From patients’ as well as GPs’ perspective, insufficient or ambiguous information from the GP influenced the decision of the non-starters to not start bisphosphonates. In contrast, starters were either properly informed, or they collected information themselves. Patients’ aversion towards medication, fear of side effects, and a low risk perception also contributed to not starting the medication, whereas starters were aware of their fracture risk and were confident of the outcome of the treatment. Concerns about osteoporosis treatment and its side effects were also expressed by several GPs. Some GPs appeared to have a limited understanding of the current osteoporosis guidelines and the indications for treatment.

**Conclusions:**

Many reasons we found for not starting bisphosphonate treatment were related to the patients or the GPs themselves being insufficiently informed. Attitudes of the GPs were shown to play a role in the decision of patients not to start treatment. Interventions need to be developed that are aimed at GPs, and at education of patients.

**Electronic supplementary material:**

The online version of this article (10.1186/s12875-018-0828-0) contains supplementary material, which is available to authorized users.

## Background

Osteoporosis is a condition that affects 1.9 per 1000 men and 16.1 per 1000 women in the Netherlands [1]. The reduced density of the bone increases the risk of fractures [2]. Fractures are associated with increased morbidity and, especially hip fractures, are associated with increased mortality for many years after the fracture [3]. Fractures at an older age are a burden on the healthcare system. A study in the Netherlands estimated that the annual healthcare costs for osteoporosis-related fractures were almost 200 Million Euros per year in 2010 [4]. It is estimated that the costs for osteoporosis-related fractures will increase with 50% between 2010 and 2030, due to an ageing population [4].

Osteoporosis can be treated with bone-sparing drugs. Bisphosphonates inhibit bone resorption, thereby reducing the risk of future fractures [5, 6]. The authors of a Cochrane review concluded that the use of alendronate can lead to a relative risk reduction of 45% for vertebral fractures, 16% for non-vertebral fractures, and 39% for hip fractures [7]. However, the effectiveness of bisphosphonates is dependent on the adherence, i.e. the extent to which patients take their medication as prescribed [8]. A meta-analysis indicated that the fracture risk increases by approximately 30% in non-adherent patients compared to adherent patients [9]. It has been shown that adherence to bisphosphonates is poor, significantly threatening the anti-fracture efficacy as well as the cost-effectiveness [10].

The process of adherence involves initiation, implementation, and continuation of medication use. Non-adherence can be the result of late or non-starting of the treatment, sub-optimal implementation of the prescribed dosing scheme, and/or early discontinuation of the treatment [11]. The reasons that patients may have for being non-adherent may be intentional or non-intentional [12]. Unintentional non-adherence is related to forgetfulness and not knowing exactly how to use medicines. In contrast, intentional non-adherence has been described as an active process, in which patients beliefs and cognition may play an important role [12].

About 30% of osteoporosis patients do not start their prescribed medication [13]. Understanding of intentional non-initiation is essential to improve the overall adherence to bisphosphonate treatment. Few studies examined the reasons that patients have for not starting with their prescribed osteoporosis medication. Main reasons include limited knowledge of osteoporosis, fear of side effects, distrust in medication in general, and a lack of belief in medication effectiveness [14–17]. Although the importance of the role of health care providers in medication adherence in general has been recognized [18], considerations of general practitioners (GPs) regarding osteoporosis medication and their awareness of patients’ intentional non-initiation have not been studied previously. Since bisphosphonates are effective in reducing fracture risk, thereby preventing serious consequences for the patients themselves and saving medical costs, it is important to thoroughly explore considerations of both patients and GPs about intentional non-initiation of bisphosphonates in patients at high fracture risk. In this study, we aimed to explore these considerations among non-starters, starters and GPs, with semi-structured interviews in order to provide a detailed overview of considerations that patients and GPs have.

## Methods

We carried out a qualitative study in which we performed semi-structured interviews, based on thematic analysis with elements of grounded theory [19]. We interviewed patients who decided not to start taking bisphosphonates despite treatment advice (intentional non-initiation), to which we will refer as non-starters. Next, we interviewed patients who did start taking bisphosphonates and continued taking them for at least three months (adherent patients, to which we will refer as starters), and asked them to reflect on the reasons that we distilled from the non-starters for not initiating treatment. In addition, GPs of non-starters were interviewed.

### Study population

Participants were recruited from a fracture prevention study and an ongoing fracture liaison service in the Netherlands. The common denominator of both is that the fracture risk assessment is centrally organised after which the GP receives the results of the evaluation and a treatment advice provided by an expert panel. From that point on, the GP is responsible for the initiation of treatment and for monitoring of the patient.

First, patients were recruited from the SALT Osteoporosis Study (SOS) [20]. The SOS is a pragmatic randomized controlled trial among women of 65 years and older in the Netherlands. It examines the effectiveness of a structured screening program and subsequent bisphosphonate treatment of patients with a high fractures risk, compared to usual care. Women with an absolute 10-years fracture risk according to the fracture risk assessment tool (FRAX) of main osteoporotic fractures including bone mineral density, and women with a prevalent vertebral fracture as determined with vertebral assessment, had an indication for bisphosphonate treatment. The treatment with bisphosphonates was initiated by the GP. By using the FRAX, the SOS protocol is less conservative than current practice in primary care in the Netherlands. Current practice according to Dutch guidelines for GPs consists of treating only patients with an increased fracture risk based on recent fracture, or several important risk factors for fractures and either low bone mineral density (*T* < − 2.5) or prevalent vertebral fracture. Therefore, more patients have an indication for bisphosphonate treatment in the SOS than in usual care. Treatment initiation was documented as part of the study by the GP. Non-starters received a written request to participate in our qualitative interview via the GP and were approached for an interview if they indicated to be willing to participate.

Secondly, patients were recruited from a transmural fracture liaison service located in the Onze Lieve Vrouwe Gasthuis hospital in Amsterdam, the Netherlands. In this fracture liaison service, the GP has a central role in initiating osteoporosis screening and initiating and monitoring subsequent treatment. The service is offered to all patients aged 50 years or older who come to the emergency department of the Onze Lieve Vrouwe Gasthuis hospital with a fracture. In consultation with the GP, patients undergo an evaluation program, including dual x-ray absorptiometry and vertebral assessment. Evaluations and treatment advices are the responsibility of an expert team. Patients identified with a high fracture risk according to the Dutch guidelines for GPs have an indication for treatment with bisphosphonates. Accordingly, the GP initiates treatment. For this study, patients received a questionnaire several months after their visit to the fracture liaison service, in which we inquired whether treatment with bisphosphonates was initiated.

Non-starters were contacted with the request for an interview. We also contacted patients for an interview who indicated to have started and continued the treatment (starters). Furthermore, we contacted the GPs of the non-starters, and asked them for an interview. Not all GPs of the interviewed patients of this study were willing to participate in an interview. We therefore contacted additional GPs from the groups of GPs participating in the SOS study who had patients who had not started bisphosphonate treatment.

### Interview procedure

The interviews with the patients were conducted face-to-face, at the patients’ home. Prior to the interview, there was no relationship between the patient and the interviewer. Before the start of the actual interview, the purpose of the interview was explained, as well as the procedure (recording of the interview and confidentiality). Semi-structured interviews were performed using a topic list (see Additional file 1:Table S1 for the final version). The topic list was based on literature and expertise of the research team, and was completed during the study in an iterative process using the data from the interviews. The interviewer specifically asked whether the GP had advised to start bisphosphonate medication. The interviews with the GPs were performed at their practice or by phone.

The interviews with starters were performed after the interviews of the non-starters had been performed and analysed. The same topic list as the topic list for non-starters was used. In addition, the starters were asked to reflect on the main reasons of non-starters at the end of the interview. The interviewer started with open-ended questions and subsequently proceeded with more specific in-depth questions.

Seven interviewers of which one GP (female), three GPs in training (1 male and 2 female) and three medical students (3 female) carried out the interviews between March 2013 and September 2016. Training of the interviewers involved practice interviews plus evaluation with the principle investigator (PE). In order to ensure optimal quality of the interviews, all interviews were analysed on content as well as competence of the interviewing techniques and discussed with the interviewer before a new interview could be performed.

### Analyses

The interviews were audio-recorded and transcribed verbatim. Transcripts were not returned to the participants for comments or corrections. The transcripts of the patient interviews were coded using the Atlas-ti qualitative data analysis software package, and the interviews with GPs were coded in Microsoft Excel 2007. Inductive analyses were performed. The interviews were analysed independently by two researchers (ED, WH, BZ, or MV). After individual coding of the interviews, the analyses were compared and discussed until consensus was reached. Disagreements were discussed with the principal investigator (PE), who also ensured the consistency of analysis method. The interviews were open-coded in the same order as the interviews were performed. The goal of open coding was to identify all aspects of the text that related to the research question. The labels of the open codes represented the text as closely as possible. In addition, these open codes were classified into main themes and subthemes. After each interview, the interview was analysed to find new themes. When there were no new themes after two subsequent interviews, we concluded that saturation was reached and ceased the interviews. The interviews of the starters were analysed to evaluated whether the reasons of non-starters were also expressed by starters, or whether they were refuted by starters.

## Results

One patient was excluded because she was sure that her GP had advised her not to start treatment. We reached saturation of data after we interviewed 16 non-starters, 13 GPs, and 10 starters. Mean age of the patients was 76.3 years, 22 out of the 26 patients were female, and 10 out of 26 patients were recruited from the fracture liaison service. Of the non-starters, only 8 had had a previous fracture, whereas all starters had a previous fracture. Among the patients without a previous fracture, one patient was identified with a vertebral fracture despite normal BMD. The mean duration of the interviews was 28:52 min for non-starters, 14:13 min for GPs and 11:12 min for starters. After analysis, we identified three main themes in the considerations patients and GPs had for not starting bisphosphonate treatment. These three themes, with subthemes, are described below.

## Main theme I: Medical advise

### Information to patients

#### Patients

Non-starters’ knowledge on osteoporosis medication was limited: most of the non-starters did not know how bisphosphonates work. Many non-starters said that they had expected to have been given more information by the GP. Starters were either well informed and had been actively seeking information themselves, or they were rather passive and followed the instructions from their GP: “*I read about osteoporosis and its treatment before the consultation”* and *“a GP knows what he talks about. If he gives me advice, I follow it*”.

Several non-starters reported that they had not fully understood the information: “*I would like to know to what extent I have it, whether it is really severe*”. One non-starter mentioned that her impression was that the GP did not understand the explanation herself. Many non-starters felt their GP easily accepted their decision and thereby approved their decision. As one non-starter pointed out: “*And what did your GP tell you? Well, he said, if you do not want to take the medication, that’s fine, it’s up to you*”. Furthermore, non-starters indicated that their GP gave mixed signals concerning whether or not to start with bisphosphonate treatment. Some non-starters said that some GPs in the end agreed with them to not start using bisphosphonates. As one non-starter explained: “*He (the GP) said, honestly, I don’t think it’s necessary for you to take the medication, I have forgotten why you should take the medication, because he said, you are a bit on the verge*”. These issues were not recognized by the starters.

#### GPs

Some GPs acknowledged that risks and details were not fully discussed during the consultation and that in some cases, the patient made a decision based on too little information. Time constraints were mentioned as reason. Some GPs mentioned to have provided mixed signals concerning treatment: “*Uhm, I have talked about my doubts concerning this intervention towards my patient, yes*”. The GPs sometimes did not question the decision of their patient not to start with the medication. The GPs gave the argument that they wish to respect the self-determination of the patient, and therefore, they would not go against the decision of their patient. Some GPs mentioned they felt unable to redirect the decision.

### Assumptions about patients

#### GPs

GPs indicated that the decision of the patient fitted with the GPs’ impression of the patients’ beliefs concerning healthcare. One GP pointed out: “*This is what I expected, so I thought to myself, I’m not going to put too much energy in this*”, or “*It makes no sense, she will stop anyway. I mean, whatever I prescribe her she will just quit*”.

## Main theme II: Medication

The theme medication concerns attitude towards preventive medication in general, attitude towards osteoporosis treatment, and side effects.

### Attitude towards medication in general

#### Patients

Many non-starters indicated that they have an aversion against medication in general, and that they would rather not take any medicine: “*I just don’t like pills, I think it’s all poison*”. Some non-starters only want to take medication that it is absolutely necessary: “*I think when you really need it, when something is life threatening, I would be okay with it*”. The starters, in general, did not express an aversion against medication, although some starters were also critical about any medication they take. Some non-starters felt confident about their own health and had a preference for lifestyle interventions: “*I believe in my own healthy lifestyle*”. Some of them also pointed out that they had a fear for side effects in general, or a distrust in the pharmaceutical industry: “*I am always afraid of side effects*”, and “*There is a commercial aspect related to the pharmaceutical industry, I mean profit seeking*”. Starters did not express a real aversion in the pharmaceutical industry. With respect to treatment duration one starter mentioned: “*I have a healthy distrust in the pharmaceutical industry. So I think that I would have checked this (the treatment duration) in due time*”.

#### GPs

Some GPs mentioned that they have an aversion against preventive medicine in general: “*I doubt every form of preventive research or preventive treatment*”.

### Attitude towards osteoporosis treatment

#### Patients

Many non-starters were unsure about the effectiveness of osteoporosis medication: “*And my mom, who had terrible osteoporosis, she had to take a lot of stuff. She still had to get a new hip and new knee, despite all the medication*”. In contrast, starters generally trusted the treatment advice they received from their GP, and expressed no distrust in the effectiveness of osteoporosis medication: “*Before the GP called me, I was already convinced that I would start using the medication*”, and: “*With this medication, you can really prevent something*”. Furthermore, some starters expressed a positive attitude towards treatment: “*It is what it is. But luckily, there is treatment available*”.

Many non-starters felt the intake instructions would result in practical problems, and they therefore preferred to take other medication such as calcium and vitamin D: “*Well, the period between taking the medication and eating is really too long for me*”, and “*The only thing I did was starting to take calcium and those vitamins*”. Some starters acknowledged that the intake of bisphosphonates is not very practical, but they got used to it, and did not see the intake instructions as a reason not to start with treatment: “*It took a lot of effort to get into the rhythm to start using the medication*”.

#### GPs

Some GPs also doubted the effectiveness of osteoporosis treatment. One GP described: “*I think all that osteoporosis, it’s very vague if I’m honest. What is meaningful, and what is not. The vitamin D, I also think that’s a difficult subject*”. Some GPs indicated that they were ambiguous about bisphosphonate treatment and its indication. “*If it’s evident a patient has osteoporosis, then you can choose to protect them, but I think the indication to treat patients is more and more expanded*”.

### Side effects

#### Patients

Many non-starters expressed fear concerning the possible side effects of bisphosphonate treatment: “*I thought, I don’t want those side effects, I already have fatigue, and I think I don’t have a very strong stomach*”, whereas starters had no fear for side effects before starting the medication: “*The information leaflet is miserable, but I just put it aside and just trust the medication*”. Several non-starters indicated that side effects experienced by relatives or friends influenced their decision not to start bisphosphonate treatment. One non-starter explained: “*I know that my cousins, my sisters, all could not endure that stuff*”. Another non-starter recalled: “*A friend of mine, she started a year ago. After the first box she said, I will stop with this mess, I’m not doing it any longer! She was done with it. She felt uncomfortable, so you have to take something that makes you feel bad, and you don’t sense that your bones are weak. Why would you take it?*”

#### GPs

Some GPs agreed with the concern of possible side effects, and expected side effects to occur with specific patients: “*My idea was, those bisphosphonates are not going to work, she cannot handle that anyway. She always has complaints of her stomach and oesophagus*”, and “*To give her bisphosphonates intravenously is also not going to work, because then she will feel sick for a year and I will be blamed for it*”. Furthermore, some GPs thought that some patients are more prone to experience side effects: “*she asked me about the side effects of bisphosphonates. I told her. Then we both thought, this is going to be a mess, we should not do this*”.

## Main theme III: Disease awareness

This theme refers to the awareness of illness, prioritizing diseases, and risk acceptance.

### Illness awareness

#### Patients

We observed a low risk perception in many non-starters. As one patient recalled: “*I thought about the facture risk in 5 or 10 years: there would be a risk, but in my opinion that was not enough to start taking medication*”. The starters were more aware of the risks of osteoporosis than non-starters: “*It shocked me that I had osteoporosis, I realised that something was wrong*”. Many non-starters indicated that they trusted their own health, and that they did not believe their diagnosis: “*I think I have pretty solid bones*”, and “*I am not convinced that I am at a higher risk than anyone else*”. Non-starters who had a fracture after a fall, did not link their fracture to osteoporosis: “*I broke my arm, my elbow, but then I had fallen from my bike. I think that even a 6-year old child with strong bones would have broken their elbow*”. Several starters, however, acknowledged that fractures they had experienced could be the consequence of osteoporosis and not just from an accident: “*I suspected that something could be wrong. If you experience two fractures within 10 years, then something is probably wrong*”. Some non-starters without fractures thought the absence of fractures was a proof of good bone health: “*I don’t think I have it, well at least not very bad, because I have never had a fracture*”.

#### GPs

GPs were not always aware of the guidelines, and therefore an increased fracture risk was not always recognized. As one GP recalled: “*The problem is not the treatment, but the diagnosis. She had a completely normal DXA result, and normal vitamin D levels. The only reason she got the diagnosis was that het risk for fractures was high was because she got a thoracic fracture due to a fall*”. This GP apparently did not know that a vertebral fracture is a very important risk factor for new fractures and an indication for bisphosphonates that is independent of bone mineral density results.

### Prioritizing diseases

#### Patients

Some non-starters expressed that they prioritized other diseases they had, and that osteoporosis treatment therefore became less important: “*And then I got the diagnosis of breast cancer, so then the conversation about osteoporosis stopped*”. The issue of prioritizing other diseases did not come up among the starters. They were more aware of their risk than non-starters and thought it was important to prevent subsequent fractures.

#### GPs

Some GPs prioritized other diseases as well: “*I thought it was much more important that she took her thyroid tablets, and she is not taking them. And that she took her acenocoumarol*”.

### Risk acceptance

#### Patients

Some non-starters indicated that they feel osteoporosis is related to aging: “*I don’t think osteoporosis is a disease, it’s just a part of getting older*”. In contrast, starters did not accept osteoporosis as just a part of getting older: “*Do you think osteoporosis is a part of getting older? Well, yes it is. But to me that is not a reason to just accept it if there are good treatment options available for it*”. Also, some non-starters rather accepted the risks and consequences of osteoporosis. As one non-starter described: “*I just take the risk to fall, then we will see what happens*”. The starters were not willing to accept the risk of a fracture: “*I thought, I am not going to take the risk that I will get another fracture*”*.* One starter also expressed: “*Being careful is always a good idea. But that does not mean you do not have to make sure your bones are as strong as possible. An accident can happen any time*” . A few non-starters indicated that they just felt too old to start osteoporosis treatment: “*I am 70 now, so how much longer do I have?”*

#### GPs

Also some GPs accepted the consequences of osteoporosis. One GP mentioned: “*So yes, the quality of life at this moment is more important than the probable prevention of fractures*”.

## Discussion

In this study, we analysed considerations of patients as well as GPs about not starting with osteoporosis treatment, despite an indication for treatment. Using semi-structured interviews, we found that these considerations focused on three main themes: medical advice, attitudes towards medication use, and disease awareness. For non-starters, insufficient information and the attitude of the GP, aversion of medication, fear of side effects, and a low risk perception contributed to non-starting their prescribed medication. Starters indicated to be properly informed, or they collected information themselves. They were aware of their fracture risk and were confident in the outcome of the treatment. For GPs, concerns about the effectiveness of osteoporosis treatment or its side effects were important considerations for not prescribing osteoporosis medication. Attitudes of the GPs were shown to play a role in the decision of patients not to start treatment.

To our knowledge, there are four studies available in which the reasons patients have for not starting bisphosphonate treatment were examined. Fear of side effects was reported as a primary reason for not starting with the medication in three of the studies [14, 15, 17]. Consistent with these previous findings, we currently observed that the fear of side effects was an important issue among non-starters. In contrast, starters were aware of the possibility of side effects, but this did not discourage them from starting the treatment. Other primary reasons that emerged in the previous studies were distrust in medication [15, 16], a low value of medication effectiveness [15, 16], and limited knowledge of osteoporosis [16]. In our study, medical advice to patients was found to be of major importance and was even identified as one of the three main themes of considerations for not starting osteoporosis treatment. Another previous study showed that fracture clinic patients reported limited understanding about osteoporosis and osteoprosis care, with ambiguity about their diagnosis, testing and treatment [21]. In our study, the purpose of the treatment was not clear for many non-starters, and many non-starters indicated to be insufficiently or ambiguously informed, whereas this was no problem for the starters. Sufficient information might also be important with respect to a low risk perception among non-starters. This observation is in line with the reduced belief compared to starters that osteoporosis is a serious disease, as observed previously [16]. In contrast to previous findings, medication costs [17] were not mentioned as primary reason for not starting bisphosphonate treatment in the current study. According to a systematic review of 24 quantitative studies, medication costs were an important factor for intentional non-initiation to any kind of medication [22]. In our study, patients did not express medication costs as a reason for not starting treatment, but this might be due to the health insurance system in the Netherlands in which most medication is reimbursed.

The currently identified considerations for non-initiation of osteoporosis treatment are very similar to previously identified considerations for non-adherence in general. Survey studies showed side effects as the most commonly reported reason to stop, but also concerns about the potential harms and motivational problems have been shown to be important [23]. A previous longitudinal qualitative study identified understanding, motivations and self-care, risk appraisal and prioritising, side effects, and decision making around medication as key themes [24]. In an overview article, the Extended Health Belief Model was used to explain medication adherence as decision making process in which perceived benefits, perceived susceptibility for and severity of fractures, concerns about or distrust in medication, medication use self-efficacy, and trust in physician are the main component [23]. The current findings fit within this framework.

In our study we explored the role of the GP in the decision about whether or not to start using osteoporosis medication. The quality of the medical consultation might be related to patient outcomes [25]. Confidence in the prescribing doctor, and trust in the healthcare system were described as important patient factors in the previous systematic review on intentional non-initiation to any kind of medication [22], and we found these factors in our study as well. From both patients’ and GPs’ perspective, we observed that GPs who legitimised the intention not to start treatment, or appeared to give advices hesitatingly, influenced their patients negatively. GPs who reported to have an aversion against osteoporosis treatment or preventive medicine in general, also had a negative influence on their patients. In addition, GPs who appeared to have inadequate knowledge about the current guidelines could not inform the patients properly. Although the current Dutch GP guideline for fracture prevention has been updated in 2012 and is applicable for several years now, not all GPs were aware of all treatment indications. Barriers to change, as often seen in changing health care settings, might be applicable. GP’s knowledge, acceptance and beliefs about bisphosphonate indication and use may form barriers. In addition, we found that the absolute fracture risk, expressed as a percentage, was often difficult to interpret for the patient as well as for the GP, and some patients felt the percentage was not high enough to start bisphosphonate treatment. The finding that patients have difficulties interpreting fracture risk was also found in another study [26]. Furthermore, a low risk perception was expressed by several patients as well as by GPs in our study and this might be partly caused by limited information on osteoporosis discussed during the medical consultation.

A strength of our study is that we performed qualitative research into considerations for not starting bisphosphonate treatment. By performing semi-structured interviews, we could generate a detailed overview of considerations, which we could not have generated if we had used quantitative data only. Besides interviewing patients who did not start treatment, we also interviewed patients who had started, and GPs of non-starters. By doing so, we were able to explore the role of the GP in starting treatment, and to analyse whether patients who did start treatment had the same or other considerations for starting treatment. Furthermore, we were able to include patients from whom the GP had not prescribed medication during consultation, but who had an indication for treatment. These patients would have been missed if non-starters would have been identified by pharmacies.

However, because of the qualitative nature of this study, our results need to be interpreted with caution and need to be confirmed in a large and non-selective sample. The study population was a self-selected population who joined either the Dutch SOS study, or were selected via a fracture liaison service. Therefore the results might not be applicable to other populations, or patients from secondary care. What additionally might have influenced our results is that there were more patients who had experienced a fracture in the sample of starters than in the sample of non-starters. This might have led to more focus on illness awareness among the starters. On the other hand, they might have been more motivated to start medication in order to prevent a next fracture. Other limitations are the relatively large number of interviewers, and the possibility that GPs mentioned other reasons behind their reluctance to prescribe to account for their behaviour.

Previous interventions to improve medication adherence were mainly patient-focussed [27]. Our study results highlight the importance of the role of the GP in the management of intentional non-initiation of osteoporosis medication. Although patient education have been shown to only marginal improve adherence [27], combined education of GP and patient might be promising, given the current observed role of the GP in shaping the view of patients. Such an approach should be examined in future research. Firstly, GPs should have a better understanding of the current osteoporosis guidelines and treatment indication, as we observed that their knowledge was not always adequate. Secondly, GPs might contribute to a more optimal osteoporosis treatment by increasing patients’ knowledge about osteoporosis and its treatment and by addressing their concerns and fears of side effects. In addition, when discussing initial fear of side effects, the GP can inform the patient that other treatment options are available, such as injectable treatment. This might help patients to make a well-informed decision about starting medication. Pharmacies might assist GPs to inform patients.

## Conclusion

In conclusion, our comprehensive qualitative study examined the considerations of both osteoporosis patients and GPs concerning intentional non-initiation of bisphosphonate treatment. Most of the factors of non-initiation were comparable to the factors that play a role in non-adherence in general. New was the way patients describe that the GP had influenced their decision, either by giving the impression that they legitimized their decision, by showing doubt, or by having insufficient knowledge. It was shown that the content of medical advice of GPs to patients and their attitudes towards medication use were primary factors. Our findings suggest that primary care might be improved by increasing both GPs’ and patients’ knowledge as well as addressing expected barriers and discussing possible solutions, in order to increase the rate of patients who start using osteoporosis medication, and thus prevent future fractures. This suggestion needs to be further examined in interventional studies. The development of such interventions, in collaboration with patients, would be the next step.

## Additional file

Additional file 1: **Table S1.** Final topic list for the interviews with patients and GPs. Topics discussed during interviews with patients and GPSs. (DOCX 13 kb)

## Abbreviations

FRAX: Fracture rate assessment tool
GP: General practitioner
SOS: SALT Osteoporosis Study
