# Supplementary material for: Factors related to intentional non-initiation of bisphosphonate treatment in patients with a high fracture risk in primary care: a qualitative study
Source: BMC Fam Pract. 2018 Aug 23;19:141. doi: 10.1186/s12875-018-0828-0 (PMC6108118; doi:10.1186/s12875-018-0828-0)
Supplement: Supplementary file 1 — Table S1. Final topic list for the interviews with patients and GPs. Topics discussed during interviews with patients and GPSs. (DOCX 13 kb) [file 12875_2018_828_MOESM1_ESM.docx]

**Table 1**. Final topic list for the interviews with patients and GPs

| Topics discussed during interviews with patients: |
| --- |
| 1. The fracture and the recovery (if a fracture had occurred) |
| 1. The contact regarding the osteoporosis screening  - medical examination - results from the examination |
| 1. The communication with the GP  - information given - advice and opinion of the GP regarding osteoporosis - treatment thoughts on medication, vitamin D and calcium - opinion of the GP |
| 1. The patient’s own attitude towards  - medication use in general - seeking information - opinions of others |
| Topics discussed during interviews with GPs: |
| 1. Beliefs of the patient regarding osteoporosis and its treatment |
| 1. Information about osteoporosis given to the patient |
| 1. Medical examination for osteoporosis and effects of bisphosphonates |
| 1. Beliefs of the GP regarding osteoporosis and its treatment |
| 1. Consultation with the patient |
| 1. Disadvantages of osteoporosis medication |
